# Insensitivity to Success and Failure: An Experimental Study of Performance-Based Feedback in Depression

**DOI:** 10.3389/fpsyg.2020.00670

**Published:** 2020-04-23

**Authors:** Huiyuan Gao, Qingqing Che, Dailin Zhang, Yinxia Chai, Xingwei Luo, Taisheng Cai

**Affiliations:** ^1^Medical Psychological Center, The Second Xiangya Hospital, Central South University, Changsha, China; ^2^Medical Psychological Institute of Central South University, Changsha, China; ^3^National Clinical Research Center for Mental Disorders, Changsha, China

**Keywords:** emotional reaction, attribution, depression, success feedback, failure feedback

## Abstract

**Objective:**

This experimental study set out to examine the effects of performance feedback (success or failure) on depressed emotions and self-serving attribution bias in inpatients suffering from major depressive disorder (MDD).

**Methods:**

The study was based on a 2 × 2 experimental design in which 71 MDD patients and 59 healthy controls participated. Both groups (MDD and controls) were randomly assigned to two conditions: success or failure in the performance feedback. A section of Raven’s Standard Progressive Matrices (SPM) was used as a bogus test of the participants’ reasoning abilities, and the Core Depressive Factor of the Zung Self-Rating Depression Scale was used to measure changes in depressed emotion in the subjects following the performance feedback. Participants then rated the accuracy of the SPM as a measure of their reasoning capacity.

**Results:**

The levels of depressed emotions in patients with MDD did not differ significantly under the two feedback conditions. In contrast, depressed emotion levels increased significantly in healthy individuals in response to failure feedback but did not change in response to success feedback. With regard to the ratings of SPM accuracy, there was no significant difference across the two feedback conditions for depressed patients; however, the accuracy ratings were higher in the success condition than in the failure condition for the controls.

**Conclusion:**

Individuals with MDD exhibit blunted emotional reactivity when experiencing new positive or negative social stimuli, supporting the theory of Emotion Context Insensitivity. In addition, self-serving attribution bias does not occur in MDD, which is consistent with the theory of learned helplessness in depression.

## Introduction

Major depressive disorder (MDD) is the most prevalent mood disorder, characterized by persistent and severe low moods, apathy, and associations with obvious suffering and functional impairment (Bora and Berk, 2016). According to the Diagnostic and Statistical Manual of Mental Disorders (fifth edition), one of the diagnostic criteria of MDD is that depressive symptoms must occur for at least 2 weeks and include deficient positive affects (e.g., anhedonia) and/or excessive negative affects (e.g., guilt, sadness). Clark et al. (1994) also suggested that patients diagnosed with MDD reported low positive and high negative affects on various questionnaires and interview measures. Durable mood disturbance is therefore considered one of the most distinguishable features of MDD (Clark et al., 1994).

The cognitive theory of depression proposed by the American psychologist Beck posited that painful childhood experiences of failure or abandonment might lead people to form a negative cognitive schema (Beck, 1967). This relatively stable and potential cognitive structure acts as a filter, allowing people to selectively notice and memorize stimuli consistent with their schema, while inconsistent information is unconsciously ignored. Bower (1981) further conceptualized the mood-congruent effect as “the enhanced effect of materials which is congruent with ongoing mood on the process of encoding and/or retrieval,” which was subsequently confirmed by a number of experimental studies (Bower, 1981; Natoli et al., 2016; Rygula and Popik, 2016). From this perspective, stimuli with a negative valence that match the persistent and low mood states of MDD patients may increase their reactivity regarding their depression level. However, Rottenberg et al. (2005) proposed the Emotion Context Insensitivity (ECI) hypothesis based on the observation that depressed inpatients exhibited very few changes in terms of expression and behavior in response to a range of environmental events. Specifically, the hypothesis suggests that mood states in MDD greatly reduce enthusiasm for activities and lead to social withdrawal behavior and a reduced emotional reactivity to new positive or negative stimuli (Rottenberg et al., 2005; Rottenberg, 2007). Further, Steele et al. (2007) found no change in reaction times to feedback information (“win” or “lose”) in depressive illness. Despite previous studies, however, the relationship between depressed emotions and positive/negative stimuli remains unclear.

Researchers have adopted the method of experimental ethology to study various emotions, and a variety of stimuli that elicit emotional responses have been proved to be a validated procedure, such as viewing pictures or videos, listening to radio programs, and engaging in certain laboratory tasks with success or failure feedback on their performance (Kayikcioglu et al., 2016; Brinkmann and Brixius, 2017). In the social sciences, feedback on performance is called performance appraisal, which reflects one aspect of social feedback. As one of the most important types of social information, Ruff and Ernst (2014) defined social feedback as comments made by others that are opinions on our personality traits or beliefs about their preference, satisfaction, and willingness to interact with us. They proposed that this feedback engages three psychological processes: anticipation, consumption, and emotion regulation. Researchers have focused more on the consumption aspect of social feedback and pointed to a lack of understanding regarding anticipation and emotion regulation (Kupferberg et al., 2016).

To examine the effect of success and failure feedback on emotion and self-recognition, a bogus test was conducted by Linville (1985); the test was described as an analytical task related to certain aspects of intelligence. Further studies of self-recognition have shown that Raven’s Standard Progressive Matrices (SPM), an achievement test in terms of intelligence without cultural or linguistic restrictions, could be used as an efficient tool in the progress of performance evaluation (Cai and Yang, 2003; Xi et al., 2007). In the current study, participants completed an SPM test and received personal feedback regarding inferior or superior performance, which was manipulated experimentally by varying the difficulty of the test. This allowed us to examine the effects of experiences with different valences on negative emotion in MDD patients and to clarify the characteristics of emotional regulation and reactivity among these patients in terms of performance-based social feedback.

Cognitive theories of emotion have asserted that individuals’ emotional responses to success and failure are governed by their beliefs about the causes of their performance. Further, the learned helplessness theory of depression (Seligman, 1975) posited that self-serving attribution bias plays an important role in mood disorders, and the suggestion has been supported by many empirical studies (Morris, 2010; Jonas et al., 2014); however, results have been contradictory in different cultural contexts (Guo et al., 2011). Self-serving attribution bias is the term used to describe the tendency to give credit to ourselves for success but attribute failure to external sources. To examine the effect of success and failure feedback on causal attribution, Dutton and Brown (1997) asked subjects to rate on a 9-point scale the validity of an accomplished achievement task in assessing integrative orientation ability. Based on their experimental design, we compared differences in self-service attribution bias across feedback conditions (success or failure) within each group (MDD or healthy controls) to explore the relationships between depressed emotion and causal attribution. Generally, our research could provide a clinically meaningful reference for identifying the best time during the course of MDD for psychotherapeutic intervention (i.e., supportive psychotherapy and cognitive behavior therapy) and for helping to stabilize patients’ conditions and promote their physical and psychological health.

Two hypotheses were tested in this study. (1) Trends for patients with MDD and healthy individuals would be different when facing social feedback of divergent valence (success or failure), supporting different theoretical models. First, findings in depressed individuals would not change with the valence of social feedback, in support of the ECI theory. Second, healthy individuals’ depressed emotions would support the mood-congruent theory, in that their depression level would decrease after receiving success feedback and increase after receiving failure feedback. (2) Healthy controls receiving success feedback would report that the test measured their real intelligence level, whereas those receiving failure feedback would not, indicating a self-serving bias in causal attribution. In contrast, the results in the depression group might support the learned helplessness theory and indicate no self-serving attribution bias after receiving any type of feedback.

## Materials and Methods

### Participants

A total of 130 Chinese participants took part in this experiment, including a depression group of 71 outpatients recruited from the Psychiatric Clinic of Xiangya Second Hospital at Central South University in China (35 male, 36 female; mean age 25.39 years, SD = 9.152). For the control group, 59 healthy participants were recruited by media advertisements (32 female, 27 male; mean age 24.85 years, SD = 8.672); these participants reported no prior or current history of depression.

The inclusion criteria for the depression group were as follows: (1) acute phase of the first episode of MDD; (2) screened with the Mini-International Neuropsychiatric Interview (MINI) and diagnosed as meeting Diagnostic and Statistical Manual of Mental Disorders (DSM)-V diagnostic criteria for MDD by a qualified clinical psychologist; and (3) total depression scores on the Zung Self-Rating Depression Scale (SDS) ≥42 (Duan, 2012). The mean SDS scores for the depression and control groups were 52.141 (SD = 6.545) and 35.085 (SD = 4.403), respectively. The exclusion criteria for both groups were: (1) psychiatric medicine use; (2) suffering from Persistent Depressive Disorder (Dysthymia) or other psychiatric disorders; (3) cognitive impairment caused by neurological disorder or other physical diseases; or (4) diagnosed with MDD in remission.

All participants signed informed consent forms, and the study was approved by the local ethics committee. General participant information is summarized in Table 1.

**TABLE 1:** Participant characteristics by group.

**Variable**	**MDD (M ± SD)**	**Control (M ± SD)**	***t*/χ^2^**	***p***
*n*	71	59		
Age, years	25.39 ± 9.152	24.85 ± 8.672	*t* = 0.347	0.729
Gender			χ^2^ = 0.161	0.688
Male	35	27		
Female	36	32		
Education level^a^			χ^2^ = 1.996	0.369
1	8	5		
2	26	16		
3	37	38		
SDS (raw score)	52.1408 ± 6.54500	35.0847 ± 4.40332	*t* = 17.668	0.000

*^*a*^Education level, 1 = junior high school and below; 2 = senior high school; 3 = undergraduate and above. SDS, Self-Rating Depression Scale.*

### Measurements

#### Mini-International Neuropsychiatric Interview

The MINI is a short, structured, diagnostic interview designed to assess diagnostic criteria according to the DSM-IV. It takes 15 min to administer, meeting the time limitations of clinical trials and epidemiological studies (Sheehan et al., 2010).

#### Zung’s Self-Rating Depression Scale

We used the 20 items of the Zung SDS as a measurement to recruit and classify participants, and the eight items of the Core Depressive Factor (items 1, 3, 6, 14, 17, 18, 19, and 20) to examine changes in depressed emotion. The Core Depressive Factor (CDF) has the greatest weight, accounting for 23.8% of the SDS variance, which mainly reflects emotional or affective symptoms of depression (Romera et al., 2008). Besides, Romera et al. (2008) reported that the Congruence Coefficient of the CDF was 0.98, which represents very high agreement according to Sakamoto et al. (1998). Additionally, Cronbach’s alpha was 0.837 for the CDF among our participants (*n* = 130), indicating a satisfactory internal consistency.

#### Raven’s Standard Progressive Matrices

Raven’s SPM is a non-verbal test that overcomes limitations of language and educational background and has been used to assess reasoning ability. Twenty problems from the SPM were selected so that the difficulty level of the test could be varied to ensure that it reflected either a success or failure condition. Half of the problems were easy (success condition), and the rest were difficult (failure condition). The difficulty level was determined on the basis of prior testing with an independent sample, and the difficulty coefficients of the easy and difficult questions in the current study were approximately 0.8 and 0.2, respectively. Each participant was asked to complete 10 questions within 5 min (Raven, 1983).

### Procedure

The study adopted a 2 × 2 mixed experimental design [(groups: MDD, healthy) × (feedback conditions: success, failure)]. At the start of the experiment, participants completed the eight items from the Core Depressive Factor of the Zung SDS, answering the questions according to their feelings at that moment. A 4-point scale was adopted, ranging from 1 (“strongly disagree”) to 4 (“strongly agree”). They were then asked to estimate how many problems (out of 10) they expected to solve correctly in the subsequent SPM test.

The reasoning test of the SPM was designed as the basic task for the entire experiment. Each participant randomly conducted Task A or Task B before receiving performance feedback, and their actual score was recorded. Task A was designed to be a successful experience, involving 10 easy problems, and positive feedback was provided irrespective of outcome. Conversely, Task B required participants to solve 10 difficult problems within 5 min and was followed by negative feedback irrespective of outcome to elicit feelings of failure. The success feedback was: “Congratulations! The results of our most authoritative intelligence test show that you have brilliant reasoning capacity, significantly exceeding 90% of your peers.” The failure feedback was: “I’m so sorry that you have failed the test. The results of our most authoritative intelligence test shows that you have poor reasoning capacity, lagging behind 90% of your peers.” Both groups of subjects were randomly assigned to the two feedback conditions. In the depression group, 39 and 32 patients were assigned to the success and failure conditions, respectively; the corresponding numbers for the healthy group were 31 and 28.

After completing the SDS and SPM, two additional questionnaires were administered to all participants. First, the eight items of the Core Depressive Factor were readministered. Second, the participants were asked to rate the accuracy of the SPM (“How accurately do you think the test assessed your actual reasoning capacity?”) on a 5-point scale ranging from 1 (“cannot detect”) to 5 (“can detect”). When they had completed these items, participants informed the experimenter that they had finished. They were then debriefed, thanked, and excused.

### Data Analysis

The collected data were analyzed using SPSS Version 21.0. Independent-samples *t*-tests were conducted to compare differences between the depression and control groups for age and the expected and actual numbers of SPM problems that participants solved. Group differences in gender and educational background were assessed with chi-square tests. Paired-samples *t*-tests were performed to compare pre- and post-test Core Depressive Factor scores and to explore the trends in depressed emotion. Analysis of variance (ANOVA) was performed to explore the effect of participant type (depressive or healthy control) and feedback condition (success or failure) on emotional reactivity. Finally, simple effects tests were performed when the interaction terms were significant.

## Results

### Descriptive Statistics

Independent-samples *t*-tests revealed no significant difference between the two groups in age or the actual number of problems solved. Regarding the expected number, the healthy group predicted that they would solve more problems than the patients with depression (*M* = 8.627, *M* = 7.648; *t* = −3.352, *p* < 0.05, Cohen’s *d* = 0.597) (Table 2). Regarding the Core Depressive Factor scores at baseline, the depression group scored significantly higher than the controls (*M* = 21.521, *M* = 13.814; *t* = 13.331, *p* < 0.01, Cohen’s *d* = 2.39). There were no group differences in gender or educational background.

**TABLE 2:** Core Depressive Factor scores and the actual and expected numbers of SPM problems solved at baseline.

**Variable**	**Depression (M ± SD)**	**Control (M ± SD)**	***t***
Core Depressive Factor	21.521 ± 3.764	13.814 ± 2.583	13.331**
Expected number^a^	7.648 ± 1.813	8.627 ± 1.449	−3.352**
Actual number^b^	6.29 ± 2.90	6.51 ± 2.84	0.159

** Statistically significant at the level of 0.01. ^*a*^Expected number = number of problems participants expected to solve. ^*b*^Actual number = actual number of problems participants solved. SPM, Raven’s Standard Progressive Matrices.

### Depressed Emotion Results

#### Differences Between Pre- and Post-Test in Depressed Emotions

For the depression group, the pre- and post-test depressed emotion scores (i.e., Core Depressive Factor scores) did not differ, regardless of the feedback condition (*t* = −0.875, *p* = 0.387; *t* = 1.408, *p* = 0.169), indicating that the performance feedback valence had little effect in subjects with MDD. For the healthy control group, pre- and post-test depressed emotion scores did not differ in the success feedback condition (*t* = 1.233, *p* = 0.231), but there was a higher post-test score in the failure condition (*M* = 13.321, *M* = 14.571; *t* = −3.35, *p* < 0.011, Cohen’s *d* = 0.44). This suggests that increased depressed emotions in the control group were only provoked by failure feedback.

#### ANOVA of Depressed Emotion

Table 3 shows that depressed emotion was potentially influenced by participant type and feedback valence. Therefore, we performed a 2 (groups: depression, healthy) × 2 (conditions: success, failure) ANOVA on depressed emotion, and the difference between pre- and post-test scores was used to compute the effect on depressed emotion. The analysis revealed a non-significant main effect for group [*F*_(1,126)_ = 1.599, *p* = 0.208] and a non-significant main effect for condition [*F*_(1,126)_ = 1.945, *p* = 0.166]. However, there was a significant group × condition interaction effect [*F*_(1,126)_ = 11.952, *p* < 0.01, Cohen’s *f* = 0.31]. Taken together, these results indicate that the differential effect of feedback condition on depressed emotion also depended on the levels of a second variable, namely, the group.

**TABLE 3:** Pre- and post-test depressed emotion scores and SPM accuracy ratings.

	**Depression**		**Control**	
	**Success (*n* = 39)**	**Failure (*n* = 32)**	**Success (*n* = 31)**	**Failure (*n* = 28)**
**Core Depressive Factor**				
Pre-test (M ± SD)	21.000 ± 3.933	21.156 ± 3.502	14.258 ± 2.032	13.321 ± 3.043
Post-test (M ± SD)	21.282 ± 4.242	21.688 ± 3.693	13.742 ± 2.569	14.571 ± 2.501
Difference (M ± SD)	−0.282 ± 2.012	0.469 ± 1.883	0.516 ± 2.350	−1.250 ± 1.974
*t*-test (pre- versus post-test)	–0.875	1.408	1.223	−3.350**
SPM accuracy	2.872 ± 1.056	2.844 ± 0.677	3.355 ± 0.798	2.607 ± 0.956

** Statistically significant at the level of 0.01. SPM, Raven’s Standard Progressive Matrices.

#### Simple Effects Tests for Depressed Emotion

Given the significant group × condition interaction, we examined two simple effects of condition: the effect of condition for the depressed group and the effect of condition for the control group. As the results of depressed emotion scores across feedback conditions within each group showed, no difference was observed between the failure and success feedback conditions for depressed individuals [*F*_(1,126)_ = 2.591, *p* = 0.112]. In contrast, for the controls, the amplitude of emotional fluctuation was larger in the failure feedback condition (*M* = −1.250) compared to the success condition [*M* = 0.516; *F*_(1,126)_ = 9.652, *p* < 0.01, Cohen’s *f* = 0.38]. Similarly, two simple effects of group were examined: the effect of group for the success condition and the effect of group for the failure condition. The results of depressed emotion scores between the two groups within each feedback condition revealed no significant difference between the depression and control groups in the success feedback condition [*F*_(1,126)_ = 2.341, *p* = 0.131]. However, in the failure condition, the amplitude of the emotional fluctuation of depressed participants (*M* = 0.469) was smaller than that of the controls [*M* = −1.25; *F*_(1,126)_ = 11.889, *p* < 0.01, Cohen’s *f* = 0.37] (Table 4). Overall, these findings suggest that participant type may have affected the feedback-related differences in depressed emotion.

**TABLE 4:** ANOVA of Core Depressive Factor scores.

**Source**	**SS**	**df**	***F***
**Participant type (A)**	**6.787**	**1**	**1.599**
Success feedback (b1)	11.003	1	2.341
Failure feedback (b2)	44.115	1	11.889**
**Feedback type (B)**	**8.256**	**1**	**1.945**
Depression (a1)	9.908	1	2.591
Control (a2)	45.889	1	9.652**
A × B	**50.735**	**1**	11.952**
Error	534.858	126	

** Statistically significant at the level of 0.01. The bolded values are independent variables. Each variable is consisted of the two levels.

### Results of Raven’s Standard Progressive Matrices Accuracy Ratings

#### ANOVA of Raven’s Standard Progressive Matrices Accuracy Ratings

To investigate the impacts of participant type and feedback condition on causal attribution, we performed ANOVA on the SPM accuracy ratings and observed a non-significant main effect for participant type. However, there was a substantial main effect for feedback condition [*F*_(1,126)_ = 6.051, *p* < 0.05, Cohen’s *f* = 0.22] and a significant group × condition interaction effect [F_(1,126)_ = 5.208, *p* < 0.05, Cohen’s f = 0.20] (Table 5). This suggests that the differential effect of feedback condition on the perceived SPM accuracy was also affected by participant type.

**TABLE 5:** ANOVA of the SPM accuracy ratings.

**Source**	**SS**	**0df**	***F***
**Participant type (A)**	**0.486**	**1**	**0.611**
Success feedback (b1)	4.030	1	4.459**
Failure feedback (b2)	0.836	1	1.247
**Feedback type (B)**	**4.819**	**1**	6.051*
Depression (a1)	0.014	1	0.017
Control (a2)	8.225	1	10.709**
A × B	**4.148**	**1**	5.208*
Error	100.353	126	

* Statistically significant at the level of 0.05. ** Statistically significant at the level of 0.01. SPM, Raven’s Standard Progressive Matrices. The bolded values are independent variables. Each variable is consisted of the two levels.

#### Simple Effects Tests of Raven’s Standard Progressive Matrices Accuracy Ratings

Given the significant interaction effect, further simple effects tests were performed. Firstly, the changes of perceived SPM accuracy rating across feedback conditions within each participant group was used to examine the simple effect of feedback condition. The results showed that there was no significant difference in perceived SPM accuracy ratings among subjects with depression across feedback conditions [*F*_(1,126)_ = 0.014, *p* = 0.017]. Conversely, perceived accuracy ratings were higher in the control group for the success condition (*M* = 3.355) than for the failure condition [*M* = 2.607; *F*_(1,126)_ = 10.709, *p* < 0.01, Cohen’s *f* = 0.40]. The differences in perceived SPM accuracy rating between the two groups within each feedback condition was then used to examine the simple effect of group. The results showed that there was no significant difference between the depression and control groups in the failure condition. In the success condition, however, healthy controls reported higher SPM accuracy ratings (*M* = 3.355) than MDD subjects [*M* = 2.872; *F*_(1,126)_ = 4.459, *p* < 0.01, Cohen’s *f* = 0.25].

## Discussion

Comparing individuals with MDD and healthy individuals at baseline, we found that the depressed emotion scores and number of SPM problems that participants expected to solve were significantly different between the two groups. Before the SPM was administered, the number of problems participants expected to solve was lower for subjects with MDD than for controls, but the actual performance was similar between groups. To rule out the possibility that education level affected the correct number of SPM problems, chi-square tests were performed to examine the between-group difference in educational background, and the tests revealed no significant difference among the three levels (junior high school and below; senior high school; undergraduate and above). In general, depressed individuals exhibited increased negative expectations regarding their personal futures compared to controls, which could be considered as hopelessness. Macleod and Salaminiou (2001) administered the Future-Thinking Task and found that depressed patients reported decreased positive expectations and fewer enjoyable experiences than healthy participants. Further, in line with the clinical manifestations of MDD (prominent and persistent low moods), the results confirmed a higher level of depressed emotion in the depression group compared to the control group.

**FIGURE 1:**
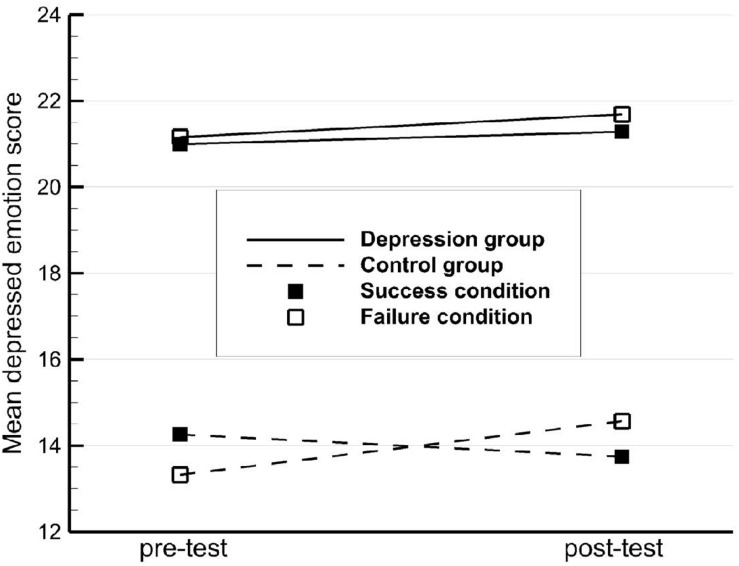
Mean depressed emotion scores by participant type and feedback condition.

ANOVA of depressed emotion scores demonstrated a significant group × condition interaction. After performing additional simple effects tests, we found in the healthy control group that the amplitude of emotional fluctuation following failure feedback increased drastically compared to that in response to success feedback. This could be explained by the cognitive dissonance hypothesis, as formulated by Leon Festinger, who proposed that cognitive dissonance causes psychological stress: cognitive dissonance refers to the existential inconsistency between two contradictory beliefs about social reality, or the contradiction between a person’s belief and an action s/he has taken (Brehm, 2010). Thus, failure feedback given by others was largely inconsistent with the self-view held by the controls, which triggered a stronger mood swing. Paired-samples *t*-tests showed that depressed emotion increased in the control group in response to failure feedback, though not to the level seen in subjects with MDD (Figure 1). This result supports the cognitive schema aspect of mood-congruent theory (Beck, 1967; Bower, 1981) and is also in line with the results reported by Zhang and Tian (2005), who performed a failure feedback experiment with 129 college students in China and found that failure feedback was effective in eliciting negative emotions (i.e., anxiety or depression). However, the results for the success feedback condition did not support our hypothesis. The reason for this discrepancy could be our measure of depressed emotion, which mainly reflected changes in negative emotions but not positive ones. Affective science, proposed by the psychologists Barrett and Russell (1998), holds that the positive and negative valences of emotion are independent of each other and are characterized by drastic bipolarity in experience and expression. Empirical research conducted by Warr et al. (1983) showed that the frequency of engaging in desirable life activities is related to increased positive emotion but not negative emotion. More recently, Ellis et al. (2009) suggested that healthy individuals reported increased positive affect after success feedback, all of which leads us to speculate that success feedback might have a greater influence on the regulation of positive emotion than on negative emotion regulation. This greater influence might explain why the depressed emotion levels of the controls were similar before and after the test in the positive feedback condition. Further investigations are required to gather more evidence about positive emotion changes in response to different valences in social feedback.

Among subjects with MDD, the amplitude of emotional fluctuation did not differ between the failure and success feedback conditions (Figure 1), favoring our prior assumption that depressed emotion in MDD subjects would not change after any type of feedback, and also favoring the ECI theory, which suggests that both positive and negative stimuli elicit reduced emotional responses in MDD patients. The finding is largely consistent with the common phenomenon experienced by MDD patients where perceptions of the world are flat and dull, and patients maintain that “everything is the same” (Healy and Williams, 1988). Empirical research conducted by Dichter et al. (2010) reported that, compared with controls, depressed individuals did not have a significant change in attitude when they viewed affective images of different valences. Additionally, a similar research in Singapore also found no significant difference between success and failure conditions among depressed patients, even with an alternative instrument of measurement (Yeo et al., 2017). Specifically, participants were asked to complete a digit-span memory task, and the Positive and Negative Affect Schedule (PANAS) was used to assess changes in negative emotion. Two consistent results predicted that the insensitivity of MDD to evaluative information from external social networks might not be associated with the instrument of measurement.

In light of these considerations, we propose that patients with MDD show blunted emotional reactivity to new stimuli with positive or negative valence. This could further explain why symptom alleviation is difficult in MDD patients experiencing an acute episode, even when they are encouraged by friends and family members around them. To optimize the therapeutic effects for patients with depressive disorders, medication is generally the first-line treatment for MDD, especially for severe cases involving suicidal thoughts. Once a patient’s condition is improved, psychological counseling or psychotherapy can be introduced (Morin et al., 2009). Stressful life events have been well recognized in previous research as precipitants of major depressive episodes. However, the presence or absence of adverse events could not provide a useful guide to the prognosis or treatment of depression. Therefore, we speculated that social feedback based on success or failure performance might have a more substantial effect on depressed patients in remission, which was confirmed by the control group in the current study. Furthermore, some psychotherapy modalities, such as supportive psychotherapy and cognitive behavioral therapy (CBT), could be more useful for patients in partial or full remission.

According to cognitive theories of emotion, emotional reactivity to success or failure outcomes is influenced by a person’s views about the causes of their performance (Weiner, 1980). We required participants to rate the accuracy of the SPM in assessing reasoning ability on a five-point scale after receiving feedback (success or failure). If the rating was high following success feedback, it meant that participants thought that the SPM test was an accurate measure of their reasoning capacity, indicating that they made internal attributions for a successful outcome. If the rating was low after failure feedback, it meant that they did not think that the SPM test was valid when assessing their reasoning ability and thus made an external attribution for the failure. Miller and Ross (1976) defined the self-serving attribution bias as the tendency to give credit to ourselves for success but attribute failure to external sources (Zuckerman, 2010).

**FIGURE 2:**
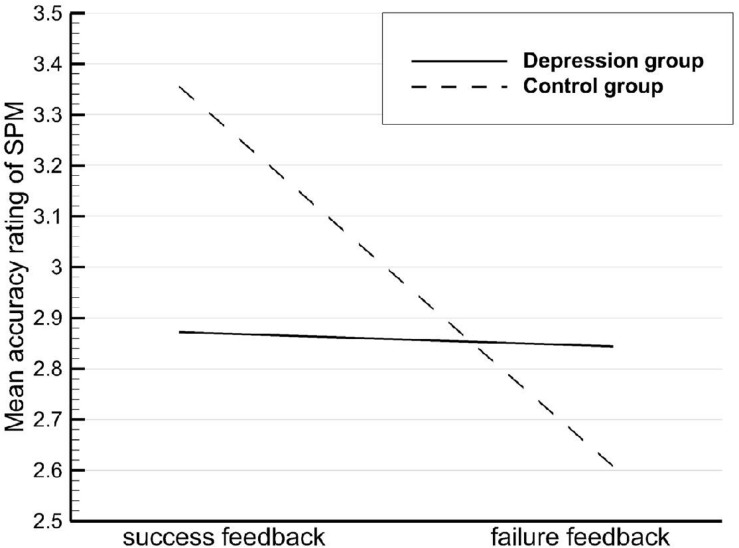
Mean Raven’s Standard Progressive Matrices (SPM) accuracy ratings by participant type and feedback condition.

The differences in self-serving attribution bias between the depression and control groups across feedback conditions were analyzed with ANOVA, and the result revealed a significant group × condition interaction effect. Using a simple effects test, we noted that SPM accuracy rating was higher following success feedback than that following failure feedback in the control group (Figure 2), indicating that healthy individuals thought that the test provided a more accurate assessment of their ability when they performed well. This result is in line with our hypothesis that self-serving attribution bias is commonly held by the general public; the result is also consistent with much of the existing literature (Streufert and Streufert, 1969; Miller, 1976). However, a meta-analysis of cross-cultural studies revealed that Westerners showed a clear self-serving bias, whereas East Asians of a relatively more collectivistic nature did not (Heine and Hamamura, 2007). The current study found that the self-serving attribution bias, which is said to be independent of collectivist culture, also occurs among the Chinese.

In the depression group, the SPM accuracy ratings were not significantly different between the two feedback conditions, suggesting that patients with MDD regarded success and failure as equally diagnostic of their ability level. Moreover, their causal attribution lacked a self-serving bias, unlike controls, which is in line with Kuiper’s findings (Kuiper, 1978). The learned helplessness model can be used to explain the attribution style of the MDD patients, characterized by a lack of difference in internal–external causal attributions in response to changes in environmental contingencies (Seligman, 1975). Thus, insensitivity to various environmental events was evident both in attribution style and emotional reactivity. Research has reported a strong correlation between causal attribution and depressive disorder (Lau and Eley, 2008; Rueger and Malecki, 2011), but how depressed patients attribute the success and failure to specific kinds of control locus (internal and external) remains unclear in the context of collectivist culture, and further research is required. A study by Fu and Chen (2010) in China suggested that individuals with depression also exhibited a self-serving bias in causal attribution, tending to take personal credit for success and blaming the external environment for failure. However, Yeo et al. (2017) proposed that patients with MDD commonly exhibit a reverse self-serving bias in causal attribution, as negative events were usually ascribed to internal, stable, and global causes. In other words, subjects with depression blamed themselves for failure but did not praise themselves for success.

Future research should also aim to rectify two major shortcomings in the current study. The first is the use of the Core Depressive Factor, which might be related to the emotional insensitivity of depressed patients to the performance-based feedback. The Core Depressive Factor has been labeled as “the affective symptoms” (Kitamura et al., 2004) and the “general depression”(American Psychiatric Association, 2013). It was used to assess the depressed emotions of patients by most researches and was hardly distinguished from emotional symptoms of depressive disorders. However, Passik et al. (2000) suggested that the emotional symptoms reflected in the core factor might be more variable and transient, and therefore less indicative of a depressive illness. Even in the case of suicidal ideation, many people express such thoughts as an indication of frustration or to “blow off steam” rather than as a genuine desire to die. Thus, to some extent, the Core Depressive Factor could be used to assess negative emotion with enriched content in healthy individuals, rather than levels of depression only. Therefore, future studies could combine the different scales. Additionally, objective physiological indicators, such as heart rate and skin electricity, could be added in order to confirm these findings.

The second shortcoming is that we did not assess the change in positive emotion after the performance appraisal. The control participants showed no difference in depressed emotions after receiving success feedback, which did not support our hypothesis. However, a previous study by Ellis et al. (2009) reported increased positive reactions in a healthy group following success feedback. We speculate therefore that positive feedback might be more effective in influencing positive emotion in healthy individuals, rather than negative emotions. Further investigations are required to gather more evidence about positive emotion changes in response to different valences in social feedback.

## Conclusion

In summary, this study has investigated the effects of performance feedback on MDD patients and healthy participants. First, the MDD patients showed little change in depressed emotions in response to both types of feedback, supporting Rottenberg et al.’s (2005) theory of ECI. Second, healthy individuals described enhanced depressed emotion in response to failure feedback, supporting the mood-congruent theory. There was no change in response to success feedback possibly because success feedback may be more effective at influencing positive rather than negative emotions. Third, self-serving attribution bias was not evident in the MDD group, supporting Seligman’s theory of learned helplessness. In contrast, self-serving attribution bias was evident in the healthy control group.

## Data Availability Statement

The datasets generated for this study are available on request to the corresponding author.

## Ethics Statement

The studies involving human participants were reviewed and approved by the Ethics Committee of Second Xiangya Hospital, Central South University. Written informed consent to participate in this study was provided by the participants’ legal guardian/next of kin.

## Author Contributions

TC and XL designed the whole study. HG and QC conceived the study. DZ, QC, and YC collected the data. HG analyzed the data and wrote the first draft of the manuscript. All authors commented on the successive drafts, contributed to the interpretation of the results, and approved the final version of the manuscript.

## Conflict of Interest

The authors declare that the research was conducted in the absence of any commercial or financial relationships that could be construed as a potential conflict of interest.
